# Thoracic endovascular aortic repair under venoarterial extracorporeal membrane oxygenation for acute aortic dissection patients: a case report

**DOI:** 10.3389/fcvm.2023.1242124

**Published:** 2023-09-04

**Authors:** Leilei Zhu, Pingping Dong, Liwen Du, Kai Xun, Peng Liu, Xiaozhen Lu, Yongwei Shi

**Affiliations:** ^1^ Emergency Department, Ningbo No. 2 Hospital, Ningbo, China; ^2^ General Medicine Department, Baihe Street Community Health Services of Yinzhou District, Ningbo, China

**Keywords:** thoracic endovascular aortic repair, V-A ECMO, aortic dissection, type A aortic dissection, case report

## Abstract

**Background:**

Open repair and replacement of the diseased aorta is still the standard treatment for type A aortic dissection (TAAD) in most patients. In endovascular treatment alone, ensuring adequate blood supply to the brain while covering the dissection with a stent is difficult.

**Case presentation:**

This study includes a 71-year-old male patient with type A aortic dissection presented at a recent follow-up examination after having undergone thoracic endovascular aortic repair (TEVAR) plus left subclavian artery chimney stent reconstruction for descending aortic dissection 5 years ago. Preoperative computed tomographic angiography, computed tomographic perfusion, and transcranial Doppler showed an intact cerebral arterial ring and good collateral circulation. We successfully performed an endovascular repair of the thoracic aorta with venoarterial extracorporeal membrane oxygenation (V-A ECMO) to protect the craniocerebral blood supply, greatly increase the safety of the operation, and ensure a good prognosis.

**Conclusion:**

TEVAR under V-A ECMO protection is beneficial for patients with TAAD because of its minimal trauma, rapid recovery, few complications, and low mortality.

## Background

Thoracic endovascular aortic repair (TEVAR) is less invasive and has a faster recovery, fewer perioperative complications, and lower mortality (1). It is effective in preventing thrombosis of the false lumen and aortic dissection enlargement, as observed in a long-term follow-up. Therefore, TEVAR has been identified as the first-line treatment for complex type B aortic dissection (cTBAD). In contrast, acute type A aortic dissection (TAAD) is usually treated with open thoracotomy and total arch replacement in the early stage, but the risks of trauma and perioperative mortality are high. This type of dissection has long been unsuitable for TEVAR (2). In recent years, the use of lumen therapy for the treatment of ascending aortic lesions has become a debated and complicated topic. In this case, we describe a patient with acute TAAD undergoing TEVAR in Ningbo Huamei Hospital, University of Chinese Academy of Sciences, under the cooperation of a vascular surgery team and an emergency ECMO team.

## Case presentation

**Figure 1:**
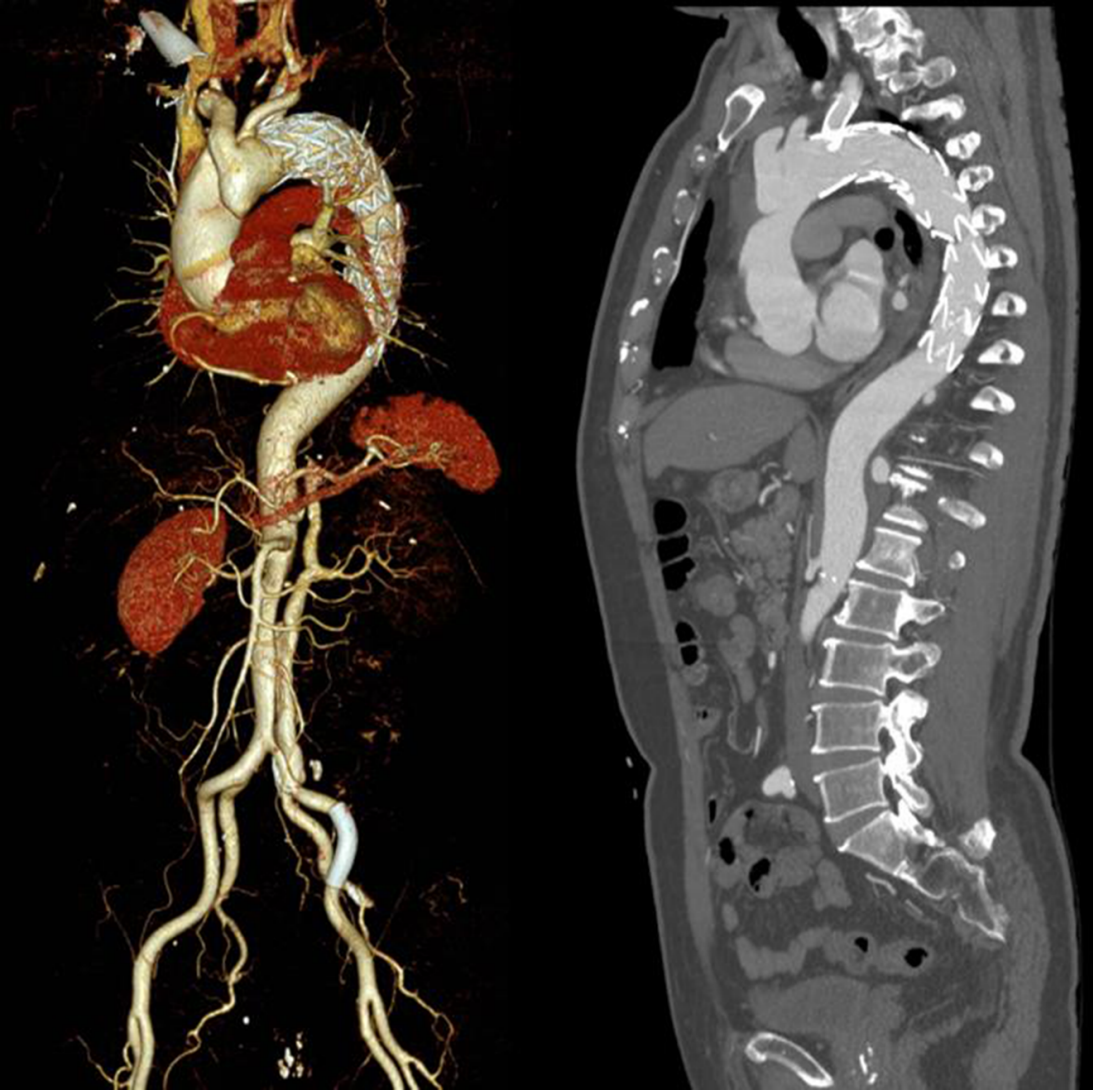
Follow up examination of aortic CTA.

A 71-year-old male patient was followed up at irregular intervals. When his chest pain symptoms gradually worsened, he was followed up in a local hospital by a CT scan, which revealed problems before coming to our hospital. TEVAR and left subclavian artery fenestration stent implantation were performed for his descending aortic dissection 5 years ago in our hospital. He had a history of rheumatoid arthritis and had been taking steroids for a long time. The follow-up computed tomographic angiography (CTA) revealed TAAD with proximal occlusion of the brachiocephalic trunk artery due to obvious compression of the false lumen (Figures 1A,B). The patient had no obvious symptoms or clinical manifestations of cerebral ischemia before surgery, and CTA, computed tomographic perfusion (CTP), and transcranial Doppler (TCD) examinations of the cerebral arteries showed that the cerebral ring was intact with good collateral circulation. After fully informing the patient of the advantages and disadvantages of thoracotomy and interventional surgery, the patient again strongly requested endovascular treatment and refused thoracotomy. After discussions between the vascular surgery team and the ECMO team, TEVAR under venoarterial extracorporeal membrane oxygenation (V-A ECMO) protection was planned. The procedure is as follows.

After successful anesthesia, routine disinfection and towel laying were performed, followed by bilateral neck and left elbow incisions to expose the bilateral common carotid artery and left brachial artery, respectively, and the proximal and distal rubber circles were retained for use. After systemic heparinization (5000 IU), bilateral carotid arteries were punctured by Seldinger’s method with an 8-F vascular sheath. The proximal innominate artery was occluded in the true lumen of the carotid artery and the innominate artery, as shown by right carotid sheath angiography. Using Seldinger’s method, we punctured the left brachial artery with a 6-F vascular sheath and inserted an indwelling pigtail catheter into the ascending aorta.

The left femoral artery was successfully punctured using Seldinger’s method, and an 11-F arterial sheath was indwelled. The right femoral vein was punctured successfully using Seldinger’s method, and a venous drainage catheter (Maquet®, 21 F) was inserted into the right atrium opening of the inferior vena cava. An arterial perfusion catheter (Maquet®, 15 F) was inserted through the right axillary artery to the proximal end of the right subclavian artery, and ECMO was successfully started at a flow rate of 1 L/min.

**Figure 2:**
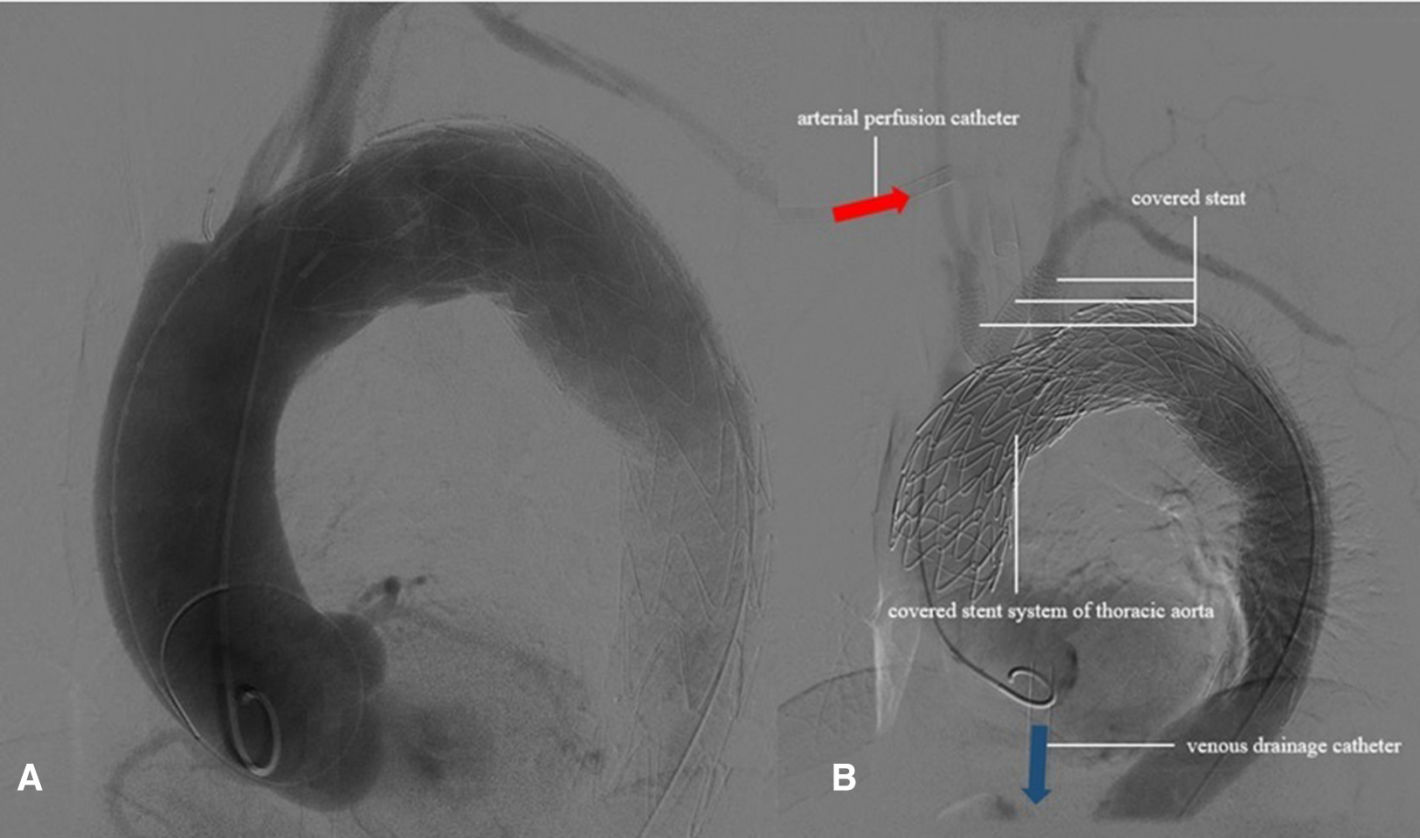
The aortic angiography showed that the dissecting aneurysm in the greater curvature of the aortic arch was formed.

The left brachial artery was punctured successfully by Seldinger’s method using a 6-F vascular sheath, followed by a pigtail catheter to the ascending aorta, and the sheath was indwelled. A 4-F labeled angiography catheter was pushed up to the ascending aorta through the left femoral artery sheath. The aortic angiography showed a dissecting aneurysm in the greater curvature of the aortic arch, involving the innominate artery, the right common carotid artery, and the right subclavian artery (Figure 2A). A covered stent (GORE® TGU454520) was inserted through the right femoral artery of the thoracic aorta and pushed upward under fluoroscopy so that the proximal marker of the stent was positioned in the ascending aorta, approximately 2 cm away from the coronary artery opening, and then released to fully cover the diseased segment.

The liver puncture needle was inserted through the vascular sheath of the left carotid artery, following the V-18 guide wire. After the successful puncture of the covered stent, the guide wire was inserted into the ascending aorta and then dilated with a 4-mm balloon. After exchanging the superhard guide wire, 6- and 10-mm high-pressure balloons were used for expansion. After swapping into the 11-F sheath, an 11 mm × 50 mm Gore VIABAHN covered stent was implanted. The proximal end exceeded the main stent of the thoracic aorta by about 1 cm, and the stent was released under fluoroscopy. In this way, the left common carotid artery *in situ* fenestration stent placement was completed. In the same way, innominate artery *in situ* fenestration stent implantation was performed by implanting a 9 mm × 50 mm Gore VIABAHN covered stent. The angiography was satisfactory.

After smooth bilateral carotid blood flow was achieved, the ECMO flow was slowly downregulated, and the whole procedure ensured that the oxygen saturation of both brains was greater than 65%. The puncture needle was inserted through the left brachial artery access, followed by a V-18 guide wire, and then the guide wire was inserted into the distal part of the descending aorta. After stepwise dilation of the puncture orifice with a balloon, the 11 mm × 50 mm Gore VIABAHN covered stent was inserted; the stent was released approximately 1 cm beyond the proximal left subclavian artery opening.

**Figure 3:**
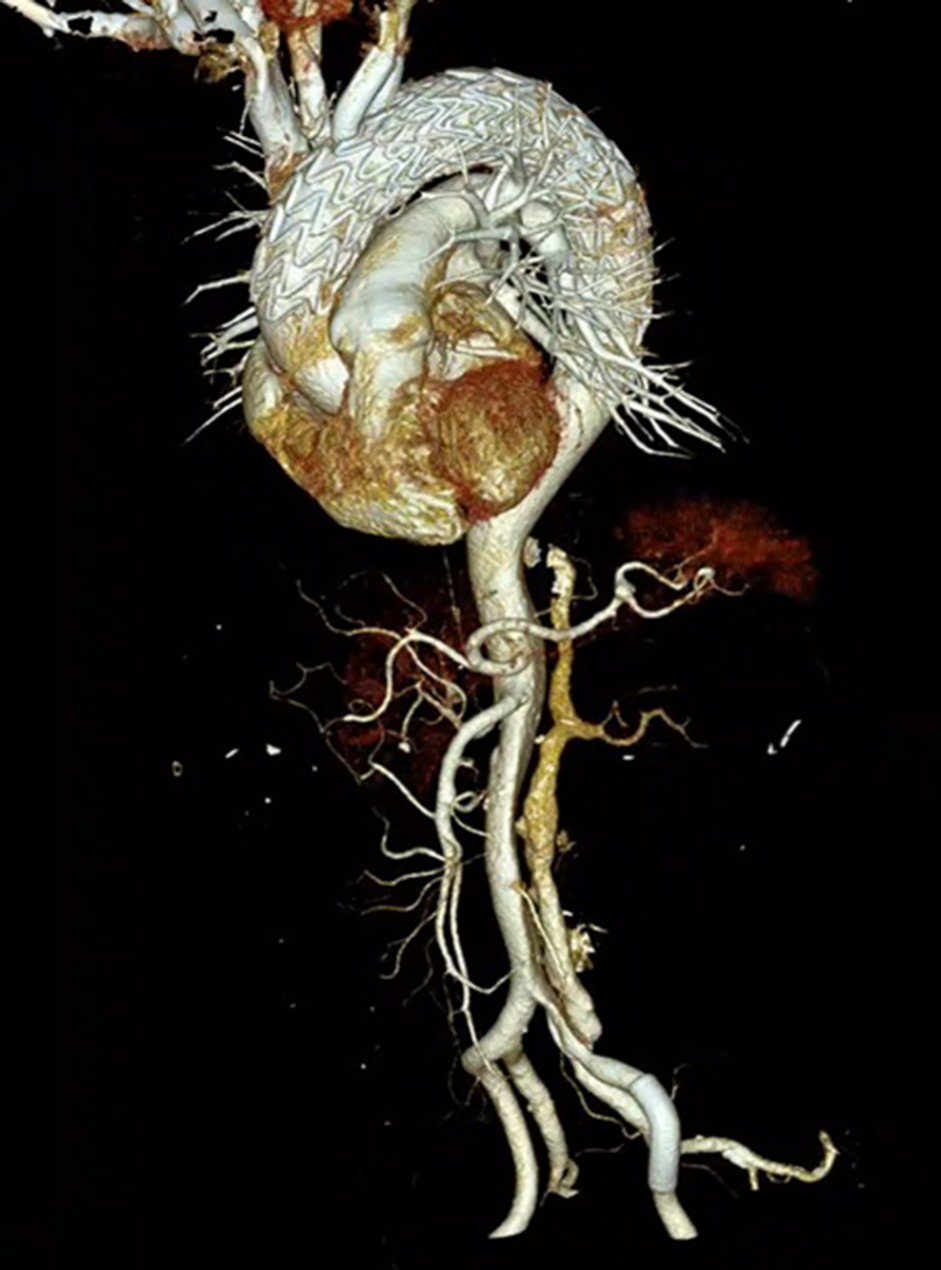
Aortography was performed at six months post-operative follow-up.

ECMO was stopped, and aortography and coronary arteriography were performed again. The results showed that the covered stent was positioned satisfactorily, the stent was close to the vascular wall, there was no twist or end leakage, and the covered stent was not blocking the coronary artery. The thoracic aortic dissection was completely isolated by covered stents, and contrast media could be seen in the innominate artery, right common carotid artery, right subclavian artery, left common carotid artery, and left subclavian artery (Figure 2B). The operation was completed, the ECMO arterial catheter was removed, and the left femoral artery puncture wound was successfully closed with an embedded ProGlide vascular suture device. The right axillary artery incision was closed with 6-0 Prolene. The right femoral vein was closed with the previously embedded ProGlide suture device, and the elastic band was applied for compression. On the second day after surgery, the patient awoke in the EICU with stable vital signs and good muscle strength in the extremities and was transferred to the ward. One week later, the patient was discharged and followed up for 6 months without obvious discomfort. CT angiography showed blood vessels in good condition (Figure 3). Informed consent was obtained from the patients and family members for case information reporting.

## Discussion and conclusions

Acute TAAD is a cardiovascular emergency caused by weakness or tear of the intima leading to the formation of a false lumen in the media. Blood rushes into the false lumen, enlarging the tear at the proximal, distal, or both ends. When the false lumen compresses the aorta, it can lead to poor perfusion of the coronary arteries, brachiocephalic trunk, and their branches, aortic valve insufficiency, and aortic rupture (3). Open repair and replacement of the diseased aorta remain the standard of care for most patients. The primary objective of surgical treatment is to remove the origin of the intimal tear and reconnect the intima with the media and adventitia to eliminate the false lumen (4). TEVAR is an additional treatment mode for thoracic aortic diseases and can be an alternative treatment for high-risk or inoperable patients. Because stent transplantation is less invasive, TEVAR is well tolerated in an older and unwell patient population without needing thoracotomy, cardiopulmonary bypass, or deep hypothermic circulatory arrest.

**Figure 4:**
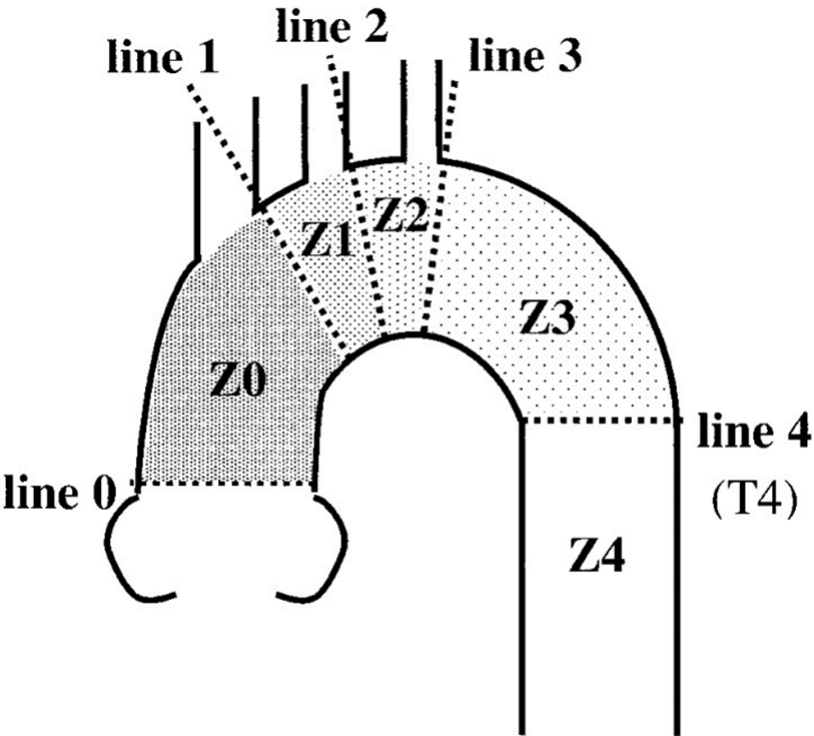
An anatomical map of each landing zone bordered by lines delineating the distal sides of the branch arteries of the aortic arch (5).

In 2002, Mitchell et al. divided the thoracic aorta into five regions ranging from 0 to 4 according to the relationship between each part of the thoracic aorta and branches of the brachial and cephalic arteries (Figure 4) (5). This case is a complex case of aortic dissection. During CTA follow-up 5 years after TEVAR for aortic dissection in the Z4 region, proximal stent dissection was found, which progressed to TAAD, involving the Z0 region and proximal occlusion of brachial and cephalic trunk arteries by obvious false lumen compression. The difficulty of the treatment lies in ensuring the blood supply to the brain while covering the dissection rupture with the covered stent, especially when the brachiocephalic trunk artery, the left common carotid artery, and the left subclavian artery are covered at the same time. Moreover, the fenestration of the three branches is not completed, the blood supply to the bilateral brain is completely blocked, and the risk of cerebral hypoperfusion is high. However, the patient refused to allow the opening of his chest due to his advanced age, poor pulmonary function, and underlying diseases. Combined with CTA, the Circle of Willis was complete, and TCD showed that the collateral circulation of the anterior and posterior cerebral communicating arteries was satisfactory and the blood flow of the jugular vein was smooth. This provides an opportunity for V-A ECMO to protect cerebral perfusion by supporting the right cerebral blood supply via right axillary artery perfusion, and the left cerebral blood supply is temporarily provided by the right cerebral blood supply via the cerebral arterial ring (Figure 3).

V-A ECMO is a well-developed and commonly performed technique to provide circulatory support to critically ill patients with refractory cardiogenic shock and cardiac arrest (6). However, due to the retrograde perfusion of the aorta, V-A ECMO has been considered relatively contraindicated in the field of aortic dissection. ECMO as a bridge to support this patient undergoing TEVAR eliminated the contraindication and provided better blood supply to the brain. The intubation strategy we adopted selected femoral vein drainage and right axillary artery perfusion, which can ensure better and more accurate flow support for the brain compared with femoral artery perfusion, and this method also prevents the femoral artery perfusion blood flow from becoming blocked by the thoracic aortic covered stent system.

Axillary artery cannulation does not require median sternotomy nor will it cause complications related to femoral artery cannulation. The literature supports that the limb can tolerate 4–6 h without a blood supply (7). In this case, the tolerable operation time of the right upper limb under ECMO protection is still uncertain. We recommend limiting the operative time or taking other measures to improve blood supply to the right upper extremity (distal arterial sheath placement to open collateral circulation).

We believe that the most important condition for unilateral cerebral artery perfusion is the integrity of the Willis ring and collateral circulation in the patient’s brain (8). In this case, the collateral circulation function of the cerebral artery ring was carefully investigated before surgery, but there was still the possibility of blood hypoperfusion in the distribution area of the left external carotid artery. At present, there is no good method to evaluate the blood supply of cervical pulp. Once high cervical pulp ischemia injury occurs, the prognosis of surgery can be seriously affected. In addition, there are no exact data on the specific flow under ECMO protection. The team estimated the possible range of cerebral blood flow based on the blood flow velocity and vessel diameter measured by TCD of bilateral common carotid arteries before surgery, which was approximately 20% of cardiac output in the calm state. We believe that in the future, intraoperative TCD monitoring of cerebral blood flow combined with cerebral oxygen monitoring can be implemented to obtain further research data.

## Data Availability

The original contributions presented in the study are included in the article/Supplementary Material, further inquiries can be directed to the corresponding author.
